# Predictors of success in left bundle branch area pacing with stylet-driven pacing leads: a multicenter investigation

**DOI:** 10.3389/fcvm.2024.1449859

**Published:** 2024-09-23

**Authors:** Ga-In Yu, Tae-Hoon Kim, Jung-Myung Lee, Daehoon Kim, Hee Tae Yu, Jae-Sun Uhm, Boyoung Joung, Hui-Nam Pak, Moon-Hyoung Lee

**Affiliations:** ^1^ Division of Cardiology, Department of Internal Medicine, Gyeongsang National University Changwon Hospital, Gyeongsang National University College of Medicine, Changwon, Republic of Korea; ^2^ Division of Cardiology, Department of Internal Medicine, Severance Hospital, Yonsei University College of Medicine, Seoul, Republic of Korea; ^3^ Division of Cardiology, Department of Internal Medicine, Sahmyook Medical Center Seoul Hospital, Sahmyook University College of Medicine, Seoul, Republic of Korea

**Keywords:** conduction system pacing, left bundle branch area pacing, stylet-driven pacing leads, interventricular conduction delay, right atrial diameter

## Abstract

**Purpose:**

Although left bundle branch area pacing (LBBAP) is an emerging conduction system pacing modality, it is unclear which parameters predict procedural success and how many implant attempts are acceptable. This study aimed to assess predictors of successful LBBAP, left bundle branch (LBB) capture, and factors associated with the number of LBBAP implant attempts.

**Methods:**

This retrospective observational multicenter study was conducted in Korea. LBBAP was attempted in 119 patients; 89.3% of patients had bradyarrhythmia (atrioventricular block 82.4%), and 10.7% of patients had heart failure (cardiac resynchronization therapy) indication. Procedural success and electrophysiological and echocardiographic parameters were evaluated.

**Results:**

The acute success rate of lead implantation in LBBAP was 95.8% (114 of 119 patients) and that of LBB capture was 82.4% (98 of 119 patients). Fewer implant attempts were associated with LBBAP success (three or fewer vs. over three times, *p* = 0.014) and LBB capture (three or fewer vs. over three times, *p* = 0.010). In the multivariate linear regression, the patients with intraventricular conduction delay (IVCD) required a greater number of attempts than those without IVCD [estimates = 2.33 (0.35–4.31), *p* = 0.02], and the larger the right atrial (RA) size, the more the attempts required for LBBAP lead implantation [estimates = 2.08 (1.20–2.97), *p* < 0.001].

**Conclusion:**

An increase in the number of implant attempts was associated with LBBAP procedural failure and LBB capture failure. The electrocardiographic parameter IVCD and the echocardiographic parameter RA size may predict the procedural complexity and the number of lead implant attempts for LBBAP.

## Introduction

Conventional right ventricular pacing (RVP) is known to cause electric and mechanical dyssynchrony, which leads to an increased risk of heart failure and mortality (1–3). Many researchers have attempted to find alternative pacing sites; conduction system pacing (CSP), which aims to directly activate the His-Purkinje conduction system and, therefore, preserve synchronous ventricular activation, is deemed to be a more physiologically similar alternative to RVP (4–6).

His bundle pacing (HBP) has been suggested as the ideal approach for physiological ventricular activation (7, 8). Nevertheless, HBP has several limitations, including technical difficulty in identifying the precise location, variable success rates, and the potential risk of premature battery depletion and lead revisions due to progressive increases in capture threshold (8, 9). In this background, left bundle branch area pacing (LBBAP), which overcomes some of the shortcomings of HBP, has been rapidly and successfully implemented in clinical practice (10). Furthermore, although most of the early LBBAP implantation and large-scale studies were conducted using lumenless pacing leads (LLLs), it has been reported that LBBAP using standard stylet-driven pacing leads (SDLs) is also available (11–13). LBBAP using SDL showed a favorable learning curve and stable pacing parameters (14). This may encourage practitioners to attempt LBBAP implantation.

As mentioned above, many studies on LBBAP have been published to date, with stable mid- to long-term results reported over the years. However, there is little research on predictors of success of the LBBAP procedure, and little is known about the impact of echocardiographic and electrocardiographic parameters on the LBBAP procedure. In addition, it is unclear how many implant attempts are acceptable without an increase in complication rate. The main purpose of this study is to investigate the echocardiographic and electrocardiographic predictors of successful LBBAP procedures using SDL. Furthermore, we report the meaningful number of lead implant attempts on LBBAP success, left bundle branch (LBB) capture success, and complication rate.

## Methods

### Study population and data collection

This is a multicenter retrospective observational study that enrolled consecutive patients who underwent LBBAP from December 2020 to February 2022 at three tertiary hospitals in the Republic of Korea. The total study population included (1) patients who underwent new pacemaker implantation (*de novo* pacemaker), (2) patients who underwent an exchange of right ventricular (RV) lead to LBBAP lead (rescue pacemaker implantation), and (3) patients who underwent LBBAP for cardiac resynchronization therapy (LBBAP-CRT or LBBAP optimized CRT). This study followed the ethical rules of the Declaration of Helsinki (2013) of the World Medical Association. In accordance with strict confidentiality guidelines, personally identifiable information was removed after the database was created. Therefore, this study was exempt from prior consent requirements.

All three centers participating in the study recorded procedure-related information, including procedure time, fluoroscopy time, and number of attempts, in the procedure record. Data collection for the study was conducted by reviewing procedure records.

### LBBAP procedural technique

LBBAP was performed with a 5.6Fr SDL with an extendable helix (Solia S60, Biotronik SE & Co KG, Berlin, Germany) delivered through a pre-shaped sheath (Selectra 3D, Biotronik). The lead was prepared as described in previous studies (14–16). In brief, the helix was extended before the procedure by turning the outer pin 10 to 12 times clockwise with a fixation-tool. After that, 5 to 10 additional clockwise turns of the outer pin using the green stylet guide tool to avoid partial unwinding of the extendable helix. To maintain the tension, the stylet was fully advanced to the tip of the pacing lead. The initial choice of sheath was to select a mid-length, mid-size curve (Selectra 3D-55-39) that was more suitable for the size of the heart. Consequently, the sheath used changes to a smaller (Selectra 3D-40-39) or larger (Selectra 3D-65-39) curve depending on the size of the patient's heart.

After the advance of the delivery sheath with the prepared pacing lead to the right ventricle (RV) over the wire, we used two approaches to locate the proper pacing site for LBBAP: (1) the His potential recording as a landmark, or (2) the simplified nine-partition method (8, 14, 17). After placing the His-RV catheter, if the His potential was recorded, the LBB area was predicted using the His bundle area as a landmark. The lead tip was placed at 1–2 cm toward the RV apex from the His area in the right anterior oblique view and perpendicular to the interventricular septum in the left anterior oblique view by counterclockwise rotation of the sheath. If the His potential was unclear or not seen because of proximal site block, we used the nine-partition fluoroscopic method to find the optimal site for LBBAP. In this method, the lead tip was directly located at the junction of the partition zone “2/5”. And when leads II and III were all positive or negative on the ECG, we aimed to move when lead II QRS to be positive and lead III QRS to be negative, preferably where a polarity discordance was identified; however, in large hearts, screwing was sometimes attempted even when both II and III were negative, at the operator's discretion. If both II and III were positive, we did not proceed due to the risk of damage to the coronary branches. When this QRS pattern was confirmed, the lead tip was advanced by fast rotation of the whole lead body 5–10 times for initial fixation. Unipolar pacing QRS morphology and impedance can be monitored at this moment, but additional turns (2–4 in every attempt) are usually needed to place the lead into the target area.

With the advancement of the lead, the LBBB pattern gradually diminished until a narrower QRS with an atypical right bundle branch block (RBBB) pattern (Qr, qR, or rSR pattern) in lead V1 was observed, suggesting that the pacing lead tip was near or at the LBB area. If LBB capture could not be demonstrated, further advancement of the lead tip was guided by monitoring the unipolar pacing impedance and/or observation of the fixation beat (18, 19). If LBB area capture could not be achieved at the initial site, the lead reposition and the number of screwing attempts were determined at the physician's discretion, but it was recommended not to try more than five times generally. The exact number of leads used was not counted, but less than 5% of all patients had more than one lead used in a single patient, as helix tips were cleaned and reused whenever possible.

During lead implantation, there is a potential risk of perforation into the LV cavity, which may result in an iatrogenic ventricular septal defect. To reduce the risk of septal perforation, a drop in unipolar impedance to <500 U and loss of myocardial current of injury were monitored.

All operators in this study had experience in implanting cardiac implantable electronic devices with conventional RV pacing but had no previous LBBAP experience. As an LBBAP strategy, there was no lumenless pacing lead and the procedure was performed only using SDL.

### Definition of LBBAP and LBB pacing (successful LBB capture)

If the left bundle branch block (LBBB) pattern gradually diminished and the RBBB delay pattern (Qr, qR, or rSR) in V1 was seen during unipolar pacing, it was considered a success for LBBAP regardless of the confirmation of LBB capture. Therefore, the LBBAP includes left ventricular septal pacing (LVSP), as well as nonselective LBB pacing (LBBP) and selective LBBP. If there was no typical RBB delay pattern in V1, whether deep septal pacing or RV conventional pacing, all were considered LBBAP failures.

LBBP is defined as a case where LBB capture is confirmed. To confirm successful LBB capture, surface ECGs (12-lead) and intracardiac electrograms were continuously monitored with an electrophysiology recording system during the procedure. The definition of successful LBB capture in this study followed and modified the definition of procedural success of LBB capture used in previous studies (20, 21). If one or more of the following findings was identified during the unipolar pacing in addition to the RBBB configuration in V1, the success of LBB capture was confirmed: (1) Abrupt changing of stimulus to left ventricular activation time (LVAT) (stimulus to peak of the R wave in V6) of >10 ms during increasing output; (2) Short and constant stim-LVAT and the shortest stim-LVAT <75 ms in non-LBBB and <85 ms in LBBB; (3) Programmed stimulation by pacing lead changes QRS morphology from nonselective LBB to left ventricular (LV) septal capture; (4) LBB potential (LBB-V interval of 15 to 35 ms); and (5) Transition from nonselective LBB capture to selective LBB capture at near-threshold outputs (When V1 showed increased R-wave peak time and broad R-wave, but V6 LVAT was constant). Examples of intracardiac electrograms obtained during the LBBAP procedure are presented in Supplementary Figure S1.

If LBB area capture could not be achieved at the initial site, operators typically aimed to try until they confirmed LBB capture. The lead reposition and the number of screwing attempts were determined at the physician's discretion, weighing the benefit of LBB capture against the risk of complications and prolonged procedure time.

### Electrocardiographic and echocardiographic variables

The 12-lead ECG data and transthoracic echocardiogram (TTE) findings of enrolled patients were collected prior to pacemaker implantation.

Classification of QRS morphology on ECG followed pre-defined criteria (22). The LBBB was defined as follows: QRS duration ≥120 ms, QS or rS in V1, and monophasic R wave without Q waves in I and V6. The RBBB was defined as follows: QRS duration ≥120 ms, rSR′ morphology in V1–V2, and a deep S wave in V6. The intraventricular conduction delay (IVCD) was defined as follows: QRS duration ≥110 ms without criteria for LBBB or RBBB.

Measurements of cardiac chambers on TTE were performed according to the American Society of Echocardiography guidelines (23). The end-systolic left atrial (LA) diameter was measured in the M mode in the parasternal long-axis view, and the end-systolic LA volume was measured in the standard four-, two-, and three-chamber views using the modified Simpson's method. The LV diameter was measured using the M mode in the parasternal long-axis view in both end-systolic and end-diastolic states. The right atrial (RA) minor axis dimension was measured in the apical four-chamber view as the distance between the lateral RA wall and the interatrial septum at the mid-atrial level, defined by half of the RA long axis. The basal RV diameter was defined as the maximal transversal dimension in the basal one-third of the RV inflow at end-diastole in the RV-focused view, and the mid-cavity RV diameter was defined as the transversal RV diameter in the middle third of the RV inflow, halfway between the maximal basal diameter and the apex (Supplementary Figure S2).

### Statistical analysis

Descriptive statistics were used to organize and interpret patient baseline characteristics and comorbidities. Categorical variables are reported as frequencies (percentages). Continuous variables are reported as the mean ± standard deviation or median with interquartile range. Categorical variables were compared using Fisher's exact test or Pearson's *χ*^2^ test, whereas continuous variables were compared using the Student's *t*-test and Wilcoxon sum-rank test.

To determine the number of meaningful attempts related to the success of LBBAP using SDL, we verified the cutoff value using MSTAT analysis. Univariate and multivariate linear regression models were used to identify electrocardiographic and echocardiographic parameters that predicted the number of attempts at lead implantation. The correlation between independent predictors and the number of trials was expressed using a scatter plot, and the strength of the correlation was expressed as Pearson's *r* value. Jittering was performed to prevent over-plotting, which can occur during continuous measurements.

All tests were two-tailed with values of *p* < 0.05, considered significant. Statistical analyses were performed using R programming version 4.0.3 (The R Foundation for Statistical Computing, Vienna, Austria).

## Results

### Baseline characteristics

A total of 119 patients were enrolled (mean age 67.7 ± 16.5 years, 47.1% female). This accounted for 13.2% (119/903) of total cardiac implantable electronic devices. The total success rate of lead implantation in LBBAP was 95.8% (114/119) for entire patients. The LBBP (successful LBB capture confirmed) and LVSP among all successful LBBAPs were 86.0% (98/114) and 14.0% (16/114). Patients with a history of valvular heart disease were 31/119 (26.1%) of all patients, and there was no significant difference between the outcome groups (*p* = 0.722). Among these, 15/31 (48.4%) patients underwent trans-catheter aortic valve implantation for severe aortic valve stenosis, and there was also no statistically significant difference between the outcomes (12.2%, 18.8%, and 0.0% in LBBP, LVSP, and LBBAP failure, respectively; *p* = 0.527). There was no significant difference in baseline QRS morphology between the group that successfully underwent LBBAP and the group that did not. Among the echocardiographic parameters, LA size and LV sizes were significantly larger in the LBBAP failure group than in the LBBAP success group. The baseline characteristics of the patients are summarized in Table 1.

**Table 1:** Baseline characteristics.

	All (*N* = 119)	LBBAP		LBBAP failure (*n* = 5)	*P* value
		LBBP^a^ (*n* = 98)	LVSP (*n* = 16)		
Demographics					
Age, years	67.7 ± 16.5	67.4 ± 17.3	69.6 ± 11.8	67.2 ± 16.6	0.89
Female sex	56 (47.1%)	51 (52.0%)	4 (25.0%)	1 (20.0%)	0.06
Body mass index, kg/m^2^	24.3 ± 3.8	24.3 ± 4.0	24.5 ± 3.1	23.4 ± 3.6	0.87
Indication for LBBAP					
Sick sinus syndrome	21 (17.6%)	15 (15.3%)	4 (25.0%)	2 (40.0%)	0.26
AV block	98 (82.4%)	83 (84.7%)	12 (75.0%)	3 (60.0%)	0.26
CRT indication	12 (10.7%)	7 (7.4%)	4 (30.8%)	1 (20.0%)	0.03
Baseline QRS morphology					0.15
Narrow QRS	51 (42.9%)	44 (44.9%)	4 (25.0%)	3 (60.0%)	
RBBB	24 (20.2%)	17 (17.3%)	6 (37.5%)	1 (20.0%)	
LBBB	21 (17.6%)	16 (16.3%)	5 (31.2%)	0 (0.0%)	
Bifascicular block	9 (7.6%)	9 (9.2%)	0 (0.0%)	0 (0.0%)	
Trifascicular block	5 (4.2%)	5 (5.1%)	0 (0.0%)	0 (0.0%)	
IVCD	3 (2.5%)	2 (2.0%)	0 (0.0%)	1 (20.0%)	
Paced rhythm	6 (5.0%)	5 (5.1%)	1 (6.2%)	0 (0.0%)	
Comorbidities					
Atrial fibrillation	35 (29.4%)	29 (29.6%)	3 (18.8%)	3 (60.0%)	0.21
Hypertension	70 (58.8%)	55 (56.1%)	12 (75.0%)	3 (60.0%)	0.36
Diabetes mellitus	31 (26.1%)	23 (23.5%)	6 (37.5%)	2 (40.0%)	0.38
Previous MI	1 (0.8%)	1 (1.0%)	0 (0.0%)	0 (0.0%)	0.90
Valvular heart disease	31 (26.1%)	27 (27.6%)	3 (18.8%)	1 (20.0%)	0.72
Congestive heart failure	45 (37.8%)	37 (37.8%)	5 (31.2%)	3 (60.0%)	0.51
Ischemic stroke or TIA	13 (10.9%)	9 (9.2%)	4 (25.0%)	0 (0.0%)	0.12
Vascular disease	25 (21.0%)	22 (22.4%)	2 (12.5%)	1 (20.0%)	0.66
Chronic kidney disease	27 (22.7%)	22 (22.4%)	3 (18.8%)	2 (40.0%)	0.61
End stage renal disease	3 (2.5%)	1 (1.0%)	2 (12.5%)	0 (0.0%)	0.02
Liver disease	5 (4.2%)	5 (5.1%)	0 (0.0%)	0 (0.0%)	0.57
Medication history					
Oral anticoagulant	36 (30.3%)	30 (30.6%)	3 (18.8%)	3 (60.0%)	0.21
Aspirin or P2Y12 inhibitor	18 (17.1%)	57 (58.2%)	13 (81.2%)	4 (80.0%)	0.15
ACE inhibitors or ARB	74 (62.2%)	16 (16.3%)	3 (18.8%)	1 (20.0%)	0.95
Beta-blockers	20 (16.8%)	1 (1.1%)	0 (0.0%)	0 (0.0%)	0.91
DHP-CCB	24 (22.9%)	21 (23.6%)	3 (27.3%)	0 (0.0%)	0.44
Non-DHP CCB	1 (1.0%)	31 (34.8%)	4 (36.4%)	3 (60.0%)	0.52
Loop/thiazide diuretics	38 (36.2%)	18 (20.2%)	4 (36.4%)	2 (40.0%)	0.31
K + sparing diuretics	24 (22.9%)	56 (57.1%)	12 (75.0%)	2 (40.0%)	0.28
Statin	70 (58.8%)	57 (58.2%)	13 (81.2%)	4 (80.0%)	0.15
Echocardiographic parameters					
LA AP diameter, mm	43.7 ± 10.4	43.2 ± 9.7	42.7 ± 6.4	56.0 ± 21.6	0.03
LA volume index, ml/m^2^	55.6 ± 46.9	52.4 ± 39.2	51.1 ± 23.0	124.7 ± 127.4	<0.001
LVEDD, mm	51.7 ± 7.0	51.2 ± 6.5	57.7 ± 14.6	55.0 ± 9.8	0.19
LVESD, mm	35.1 ± 8.3	34.5 ± 7.4	47.0 ± 19.1	36.8 ± 10.1	0.03
RA minor axis diameter, cm/m^2^	2.2 ± 0.5	2.1 ± 0.5	2.2 ± 0.6	2.5 ± 0.6	0.29
RA major axis diameter, cm/m^2^	2.9 ± 0.6	2.9 ± 0.6	2.9 ± 0.6	3.4 ± 1.0	0.21
RV basal diameter, cm	2.0 ± 0.4	2.0 ± 0.4	2.1 ± 0.4	2.2 ± 0.6	0.40
RV mid-cavity diameter, cm	1.5 ± 0.3	1.5 ± 0.3	1.5 ± 0.4	1.6 ± 0.4	0.68
LVEF,%	60.0 ± 15.4	61.7 ± 14.0	49.2 ± 18.1	54.2 ± 24.5	0.02
E/E’	14.8 ± 7.7	13.7 ± 6.2	18.1 ± 3.9	24.4 ± 19.6	0.01

Values are presented as mean ± standard deviation or *n* (%).

LBBAP, left bundle branch area pacing; LBBP, left bundle branch pacing; LVSP, left ventricular septal pacing; SSS, sick sinus syndrome; AV block, atrioventricular block; CRT, cardiac resynchronization therapy; RBBB, right bundle branch block; LBBB, left bundle branch block; IVCD, intraventricular conduction delay; MI, myocardial infarction; TIA, transient ischemic attack; ACE, angiotensin-converting enzyme; ARB, angiotensin II receptor blocker; DHP, dihydropyridine; CCB, calcium channel blocker; LA, left atrium; AP, anteroposterior; LVEDD, left ventricular end-diastolic diameter; LVESD, left ventricular end-systolic diameter; RA, right atrium; RV, right ventricle; LVEF, left ventricular ejection fraction.

^a^ When LBB capture was observed, it was confirmed to be LBBP.

### Procedural and electrophysiological characteristics

Patients with failed LBBAP had a significantly higher number of attempts for lead implantation than patients with successful LBBAP (3.8 ± 1.3 vs. 2.1 ± 1.1; *p* = 0.002). The time required for the procedure was significantly longer in patients with failed LBBAP than in patients with successful LBBAP. As a methodology for finding the optimal initial pacing site using a pre-shaped sheath, there was no relationship between the result of the procedure and the method of landmarking the His area, i.e., using electrocardiogram or the 9-partition method on fluoroscopy. The LBBAP failure group had a longer QRS duration on the electrocardiogram (ECG) than the LBBAP success group (146.0 ± 30.2 vs. 126.63 ± 30.47 ms; *p* = 0.424). In the successful LBBP, the LBB capture threshold at implant was 0.9 ± 0.9 V, sensed R wave amplitude was 10.0 ± 4.3 mV and pacing impedance was 680.0 ± 105.2 *Ω*. Procedural and electrophysiological characteristics are summarized in Table 2.

**Table 2:** Electrophysiological characteristics and procedural characteristics.

	All (*N* = 119)	LBBAP		LBBAP failure (*n* = 5)	*P* value
		LBBP^a^ (*n* = 98)	LVSP (*n* = 16)		
Right subclavian venous access	7 (6.7%)	4 (4.5%)	2 (18.2%)	1 (20.0%)	0.11
Methods for the location of the proper initial pacing site					
His potential recording as a landmark	37 (32.5%)	33 (34.7%)	2 (13.3%)	2 (50.0%)	0.19
Simplified 9-partition method	77 (67.5%)	62 (65.3%)	13 (86.7%)	2 (50.0%)	0.19
Number of attempts, *n*	2.2 ± 1.2	2.0 ± 1.1	2.7 ± 1.2	3.8 ± 1.3	<0.001
Number of sheath changes, *n*	0.3 ± 0.6	0.3 ± 0.6	0.4 ± 0.6	1.0 ± 0.8	0.02
Fluoroscopy time, min^b^	15.0 ± 11.3	13.2 ± 7.8	13.9 ± 8.5	51.2 ± 10.3	<0.001
Procedure time, min^b^	63.5 ± 22.2	60.3 ± 20.0	72.4 ± 17.4	111.8 ± 15.9	<0.001
Pre-implant QRS, ms	126.2 ± 30.0	124.7 ± 29.8	138.2 ± 33.2	115.6 ± 15.0	0.18
post LBBAP QRS, ms	123.8 ± 22.3	120.5 ± 19.6	139.4 ± 27.3	146.0 ± 30.2	<0.001
Sensed R wave amplitude, mV	10.1 ± 4.5	10.0 ± 4.3	11.9 ± 5.8	8.8 ± 2.7	0.38
Ventricular capture threshold, V	0.9 ± 0.8	0.9 ± 0.9	0.8 ± 0.2	0.7 ± 0.3	0.83
Ventricular pacing impedance, *Ω*	675.6 ± 109.6	680.0 ± 105.2	693.6 ± 110.9	531.6 ± 110.9	0.01

Values are presented as median (quartiles), mean ± standard deviation, or *n* (%).

LBBAP, left bundle branch area pacing; LBBP, left bundle branch pacing; LVSP, left ventricular septal pacing; LBB, left bundle branch; Stim-LVAT, pacing stimulus to left ventricular activation time.

^a^ When LBB capture was observed, it was confirmed to be LBBP.

^b^ Except rescue pacemaker implantation and cardiac resynchronization therapy.

### Procedure outcomes and number of attempts for LBBAP

The total success rate of lead implantation in LBBAP using SDL was 95.8% (114/119) for all patients. The LVSP and LBBP of all successful LBBAPs were 86.0% (98/114) and 14.0% (16/114).

**Figure 1:**
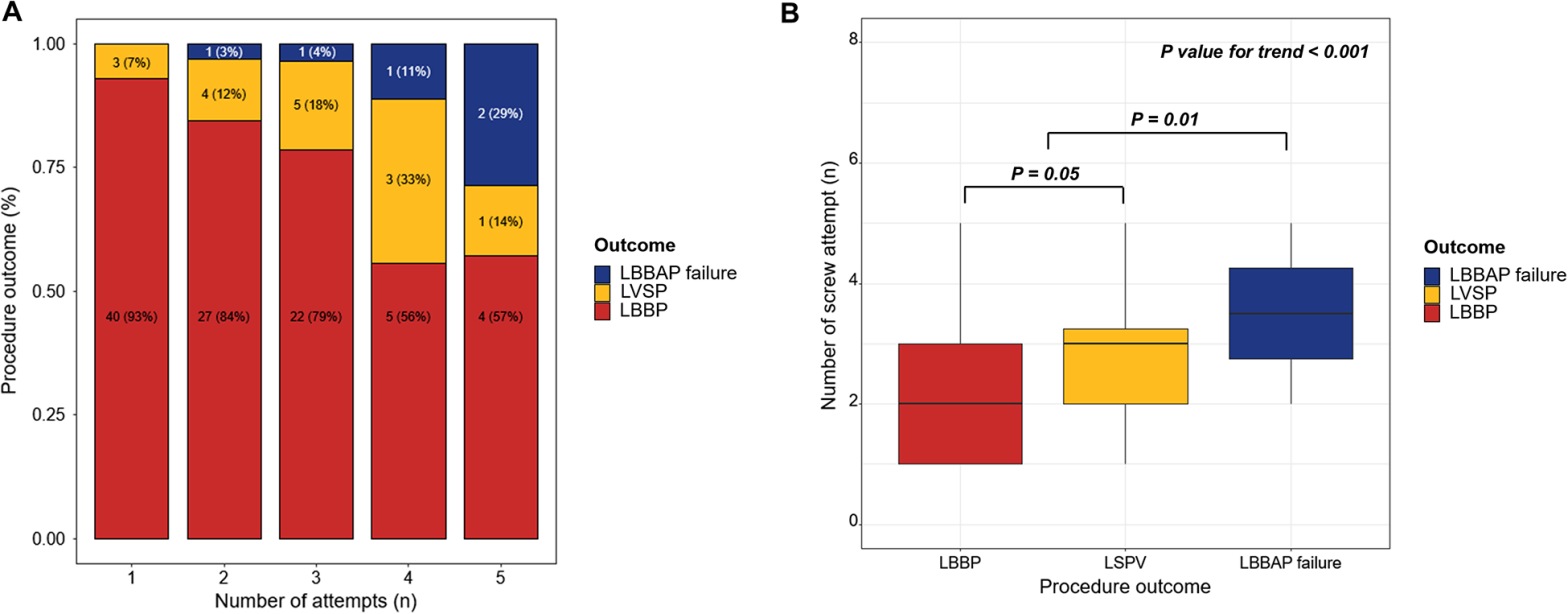
Acute procedural outcomes of the LBBAP using SDL according to the number of attempts. **(A)** The greater the number of attempts at LBBAP using SDL, the more likely the procedure was to fail **(B)** There was a significant correlation between the procedural outcome and number of attempts. LBBAP, left bundle branch area pacing; LVSP, left ventricular septal pacing; LBBP, left bundle branch area pacing; SDL, stylet-driven pacing lead.

The greater the number of screw attempts for LBBAP lead implantation, the more failures that occurred (Figure 1A). The number of attempts increased significantly with LVSP and LBBAP failures over LBBP (*p* < 0.001). In the inter-group comparison, there was a significant difference between LBBP and LVSP (2.0 [1.0–3.0] in LBBP vs. 3.00 [2.00–3.50] in LVSP; *p* = 0.05) and LBBAP and LBBAP failure (2.0 [1.0–3.0] in LBBAP vs. 4.00 [3.00–5.00] in LBBAP failure; *p* = 0.01) (Figure 1B). As a result of evaluating the cutoff value between the relationship between LBBAP outcome and number of attempts through MSTAT analysis, attempts up to 3 were meaningful trials related to the success of the procedure (*M* = 3.1046, *p* = 0.01) (Supplementary Figure S3). When comparing procedural outcomes between patients with three or fewer attempts and those with four or more attempts, there was a significant difference in the procedural success rate between the two groups (98.06% in three or fewer vs. 81.35% in over three attempts) (Table 3).

**Table 3:** Effect of the number of attempts on procedural outcomes.

	Three or fewer times (*n* = 103)	Over three times (*n* = 16)	*p*-value
LBBAP capture subtypes			0.002
LBBP	89 (86.41%)	9 (56.25%)	
LVSP	12 (11.65%)	4 (25.00%)	
Success of the LBBAP			0.014
LBBAP (LBBP + LVSP)	101 (98.06%)	13 (81.25%)	
Failed LBBAP	2 (1.94%)	3 (18.75%)	
Complication			
Procedure related complication	5 (4.85%)	3 (18.75%)	0.126
Lead related complication	3 (2.91%)	2 (12.50%)	0.268

LBBP, left bundle branch pacing; LVSP, left ventricular septal pacing; LBBAP, left bundle branch area pacing.

The initial choice of sheath was to select a mid-length, mid-size curve and during the procedure, the sheath was exchanged for a smaller or larger sheath at the operator's discretion. When the RA size of enrolled patients was divided into quartiles, a large final sheath was needed in the group with large RA size (Supplementary Figure S4).

**Figure 2:**
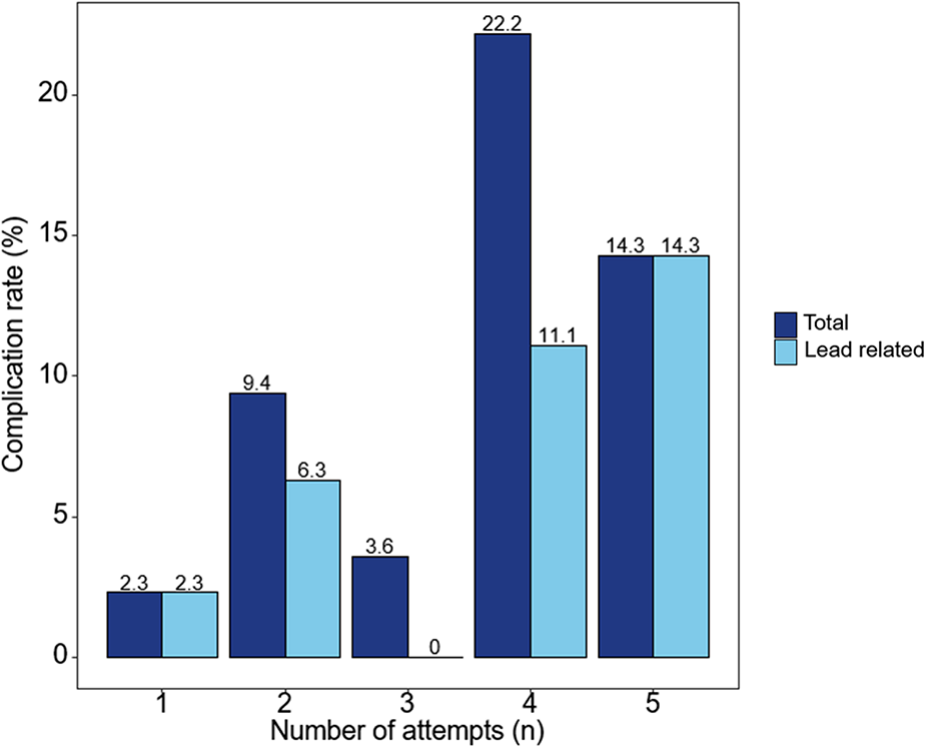
Complications of the left bundle branch area pacing using a stylet-driven pacing lead according to the number of attempts. As the number of lead implant attempts increases, the tendency for acute procedural complications increases.

The total number of complications was eight (6.7%), of which five (4.2%) were related to the pacing lead. There were three cases of LBBAP lead dislodgement. Among the lead dislodgements, one occurred during the procedure, one on the day after the procedure, and one 6 months after the procedure. There were three other procedural-related complications which included one ventricular septal hematoma, one iatrogenic ventricular septal defect, and one pocket hematoma requiring prolonged hospitalization. The ventricular septal hematoma was absorbed during follow-up, and the ventricular septal defect is being followed up through TTE to determine long-term effects. There was a trend toward increasing acute procedural complications as the number of attempts increased (Figure 2); however, this was not statistically significant (Table 3).

### Predictors of successful LBBAP using SDL

Logistic regression was performed to evaluate the factors related to the success of LBBAP (Supplementary Table S1). Baseline QRS morphology IVCD was statistically significantly associated with the success of LBBAP [odds ratio (OR) = 0.76 (0.60–0.96), *p* = 0.02]. There were no baseline QRS morphology or echocardiographic parameters significantly associated with the success of LBBP. Among procedural characteristics, number of attempts for lead implantation was related to both LBBAP [OR = 0.97 (0.95–1.00), *p* = 0.04] and LBBP [OR = 0.94 (0.89–0.99), *p* = 0.02].

**Figure 3:**
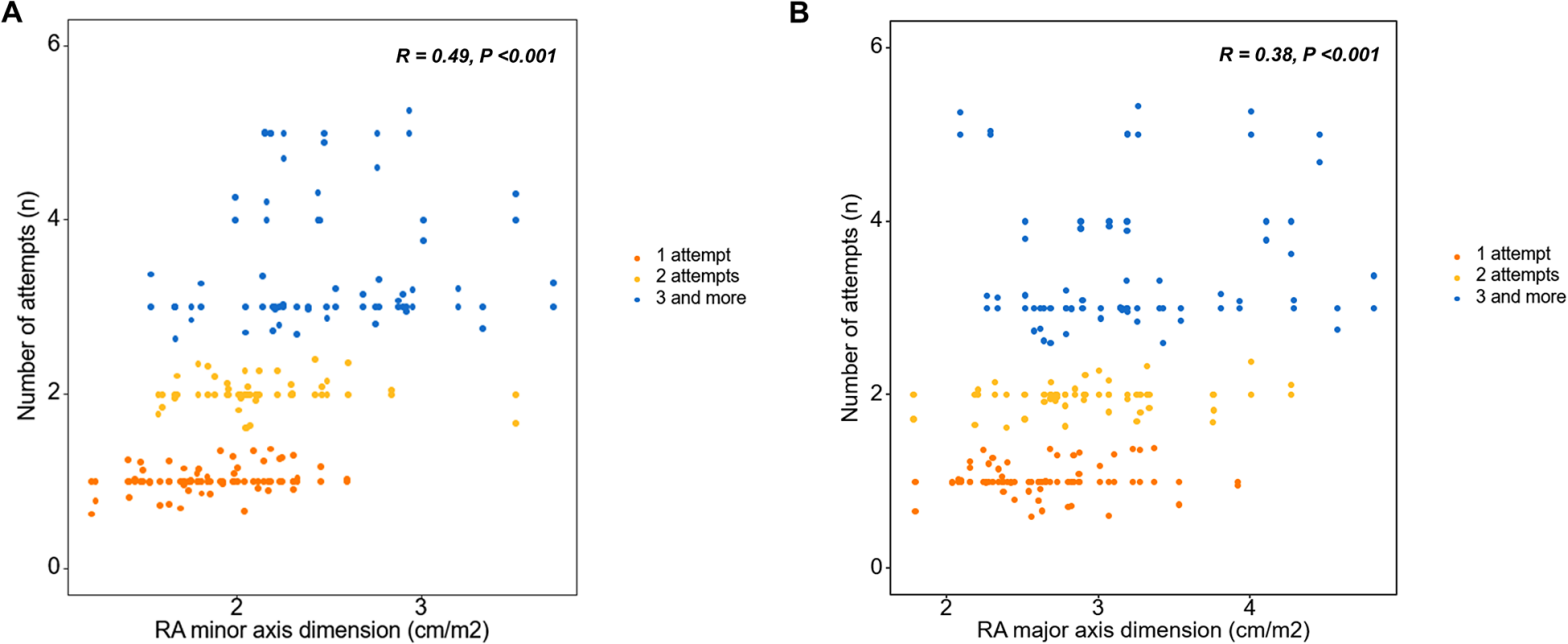
Right atrial chamber size and number of attempts for the left bundle branch area pacing using a stylet-driven pacing lead. **(A)** A larger RA minor axis dimension and **(B)** larger RA major axis dimension were correlated with more procedure attempts. RA, right atrial.

Linear regression was performed to evaluate the factors related to the number of lead implantation trials for LBBAP. In the univariate linear regression, the IVCD on ECG and LA anteroposterior diameter, LV end-diastolic diameter, RA minor axis dimension, RA major axis dimension, and RV basal dimension on TTE were related to the number of attempts (Table 4). In the subsequent multivariate linear regression for ECG variables with echocardiographic parameters, the IVCD showed a significant correlation with the number of attempts [Estimates = 2.33 (0.35–4.31), *p* = 0.02] (Table 4). In the multivariate linear regression with ECG parameters for the echocardiographic variables, the RA minor axis diameter was significantly related to the number of attempts [Estimates = 2.08 (1.20–2.97), *p* < 0.001] (Table 4). A scatter plot was used to determine the degree of relevance; the Pearson correlation coefficient of the RA minor axis dimension was 0.49 (Figure 3A) and RA major axis dimension was 0.38 (Figure 3B).

**Table 4:** Linear regression for predictors of the number of attempts for LBBAP.

	Unadjusted estimate (95% CI)	*p*-value	Adjusted estimate, model 1^a^ (95% CI)	*p*-value	Adjusted estimate, model 2^b^ (95% CI)	*p*-value
Electrocardiographic characteristics						
Pacing indication						
Sick sinus syndrome	0.51 (−0.05 to 1.06)	0.07				
Complete AV block	−0.13 (−0.56 to 0.30)	0.55				
Baseline QRS morphology						
Reference; Narrow QRS						
RBBB	−0.17 (−0.75 to 0.41)	0.55				
LBBB	0.07 (−0.54 to 0.68)	0.82				
Bifascicular block	−0.33 (−1.17 to 0.52)	0.45				
Trifascicular block	−0.22 (−1.31 to 0.88)	0.70				
IVCD	1.45 (0.06 to 2.84)	0.04	2.33 (0.35–4.31)	0.02		
Pacing rhythm	0.12 (−0.89 to 1.13)	0.82				
Echocardiographic parameters						
LA AP diameter, mm	0.03 (0.01–0.05)	0.01			−0.02 (−0.05 to 0.01)	0.20
LVEDD, mm	0.04 (0.01–0.08)	0.02			−0.01 (−0.06 to 0.03)	0.63
RA minor axis diameter, cm/m^2^	1.15 (0.75–1.54)	<0.001			2.08 (1.20 to 2.97)	<0.001
RA major axis diameter, cm/m^2^	0.70 (0.36–1.04)	<0.001			0.07 (−0.58 to 0.73)	0.82
RV basal diameter, cm	0.62 (0.04–1.21)	0.04			−0.84 (−1.70 to 0.03)	0.06
RV mid-cavity diameter, cm	0.54 (−0.12 to 1.19)	0.11				
LVEF,%	−0.02 (−0.04 to −0.01)	<0.001			−0.01 (−0.03 to 0.02)	0.56

CI, confidence interval; RBBB, right bundle branch block; LBBB, left bundle branch block; IVCD, intraventricular conduction delay; LA, left atrium; AP, anteroposterior; LVEDD, left ventricular end-diastolic diameter; LVESD, left ventricular end-diastolic diameter; RA, right atrium; RV, right ventricle; LVEF, left ventricular ejection fraction.

^a^ The model 1 was adjusted for age, sex, pacing indication, right subclavian venous access, simplified 9-partition method for initial lead position, and echocardiographic parameters.

^b^ The model 2 was adjusted for age, sex, pacing indication, right subclavian venous access, simplified 9-partition method for initial lead position, and baseline QRS morphology.

## Discussion

With the increasing importance of physiological pacing, LBBAP has recently become a popular CSP strategy. LBBAP has the advantage of providing a more stable capture threshold compared to HBP and achieves a similar paced QRS duration (24). The long-term efficacy and safety of LBBAP have been proven previously (18, 25). Moreover, the indications are expanding to include not only simple pacing but also resynchronization strategies (26, 27). Most of these early trials and studies were conducted through LBBAP using LLL. However, LBBAP using SDL, which is conventionally used for RVP, is not only possible but also shows comparable results to LBBAP using LLL (11, 15). LBBAP using SDL requires fewer cases to learn the procedure compared to those for HBP or even LBBAP using LLL (14, 24, 28). While sufficient evidence for LBBAP as a CSP strategy has been secured, there has been little research on the predictors of procedure success. In particular, there are even fewer reports on the predictors of LBBAP success using SDL. This study aimed to evaluate whether electrocardiographic and echocardiographic features could predict the success of LBBAP using the SDL procedure. Furthermore, we aimed to establish evidence for identifying optimal candidates for LBBAP, reducing the number of unnecessary lead implant attempts, and reducing procedural time and complications.

### Number of attempts for LBBAP using SDL and procedure outcome

In this study, the success rate of LBBAP was 95.8%, of which the success rate of LBBP was 86.0%. The success of both LBBAP and LBBP was related to the number of lead implantation attempts. There was a high incidence of procedural failure in patients with more attempts of LBBAP lead implantations. This implies that indefinite attempts do not necessarily guarantee success. We found that up to three attempts were associated with success and that more than four attempts were often not fruitful. Additionally, although not statistically significant, there was a trend toward more procedure-related complications with a higher number of attempts.

### Electrocardiographic and echocardiographic predictor for LBBAP using SDL

In the univariate linear regression, the electrocardiographic parameter associated with the number of attempts was IVCD. In multivariate analysis adjusted for echocardiographic variables, patients with an IVCD required more attempts for LBBAP lead implantation using an SDL than did patients without an IVCD. IVCD, which can be usually accompanied by cardiomyopathies, is not a typical conduction system disorder and the proximal conduction system is usually preserved (29). Because LBBAP targets the conduction system at an anatomically distal level rather than HBP, which could potentially be attributed to the failure of deep lead deployment due to progressive fibrosis in the septal myocardium in LBBAP. Therefore, CSP cannot correct QRS prolongation in IVCD. IVCDs have also been reported as predictors of LBBAP using LLL (30).

Right heart chamber size (RA minor axis diameter, RA major axis diameter, and RV basal diameter) were related to the number of attempts. Multivariate analysis adjusting for echocardiographic variables, confirmed that the RA minor axis was significantly related. The larger the RA minor axis, the greater the number of attempts, and a statistically significant correlation was confirmed between the RA minor axis and the number of attempts (Pearson *R* = 0.49, *p* < 0.001). RA size, an echocardiographic parameter, predicted the success of the procedure itself, and not the long-term outcome of the procedure. In this study, a larger right atrium required more procedural attempts and was associated with procedural failure. LBBAP is a procedure in which lead implantation is inevitably performed with sheath backup, and the sheath is located from the right atrium to the right side of the interventricular septum. Although commercially available sheaths for LBBAP are standardized in various sizes, the length and angle are limited, therefore they cannot fit all sizes of cardiac chambers. Therefore, if the size of the right atrium is large, sheath backup may be inadequate and it is considered that this may have an effect on the outcome of the procedure.

### Study limitations

This study had certain limitations. First, this was a retrospective, observational, non-randomized study. Second, all operators from all institutions participating in this study experienced LBBAP for the first time. Therefore, the results cannot be generalized because patients within the physician's learning curve period were included. Third, the number of patients with LBBAP implant failure was small. This means that LBBAP as a CSP strategy has a wide range of uses, but this study was limited because it analyzed only the success or failure of the procedure. Additionally, the small number of failures made it difficult to identify predictor of outcomes and led to indirect analysis through the number of attempted procedures. There are many other variables that may be associated with multiple lead implant attempts, such as the operator's perception of the beneficial effect of successful LBB capture, the patient's indication for the procedure, and the presence or absence of scarring in the septum. However, due to the limited number of patients in this study, we were unable to identify these factors. Fourth, this analysis evaluated the implant success of the LBBAP procedure, but long-term clinical outcomes were not evaluated. Follow-up is needed to determine whether intrinsic electrocardiographic and echocardiographic parameters are related to long-term outcomes. Fifth, among the patients enrolled in the study, the number of patients with previous myocardial infarction was very small. This was because of the difficulty of the procedure due to septal scar and fibrosis, which may have resulted in selection bias.

## Conclusion

A greater number of lead implantation attempts was associated with unsuccessful LBBAP using SDL. In the LBBAP using the SDL procedure, attempting lead implantation more than three times did not increase the success rate. It may be helpful to review the electrocardiogram and echocardiographic findings before the procedure, and the relevant parameters related to the number of procedure attempts are the IVCD and RA minor axis dimensions.

## Data Availability

The raw data supporting the conclusions of this article will be made available by the authors, without undue reservation.
