# Supplementary material for: Predictors of success in left bundle branch area pacing with stylet-driven pacing leads: a multicenter investigation
Source: Front Cardiovasc Med. 2024 Sep 23;11:1449859. doi: 10.3389/fcvm.2024.1449859 (PMC11456445; doi:10.3389/fcvm.2024.1449859)
Supplement: Supplementary file 1 [file Datasheet1.docx]

**SUPPLEMENTAL MATERIAL**

**Supplementary Figure S1.** Examples of intracardiac electrograms during the left bundle branch area pacing.

**
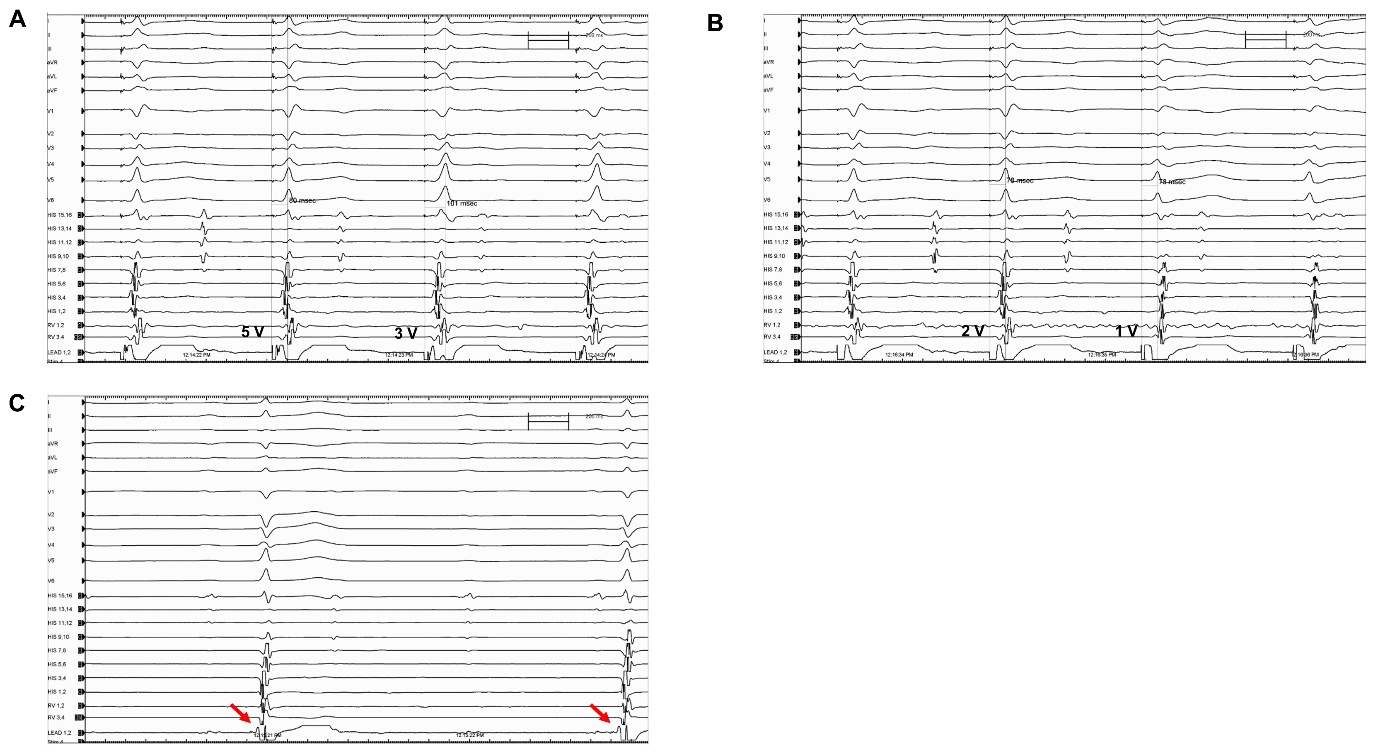
**

(A) After initial penetration with 10 rapid rotations of whole lead body, unipolar pacing (5 V to 3 V) reveals transition from nonselective capture to left ventricular myocardial capture. (B) After further advancement of lead tip by one or two rotations, shortened pacing stimulus to peak left ventricular activation time (at 2 V) and maintained at low output pacing (at 1 V) is shown. (C, red arrow) Left bundle branch potential is seen.

V, volts.

**Supplementary Figure S2.** Methods for right-sided cardiac chamber size quantification.


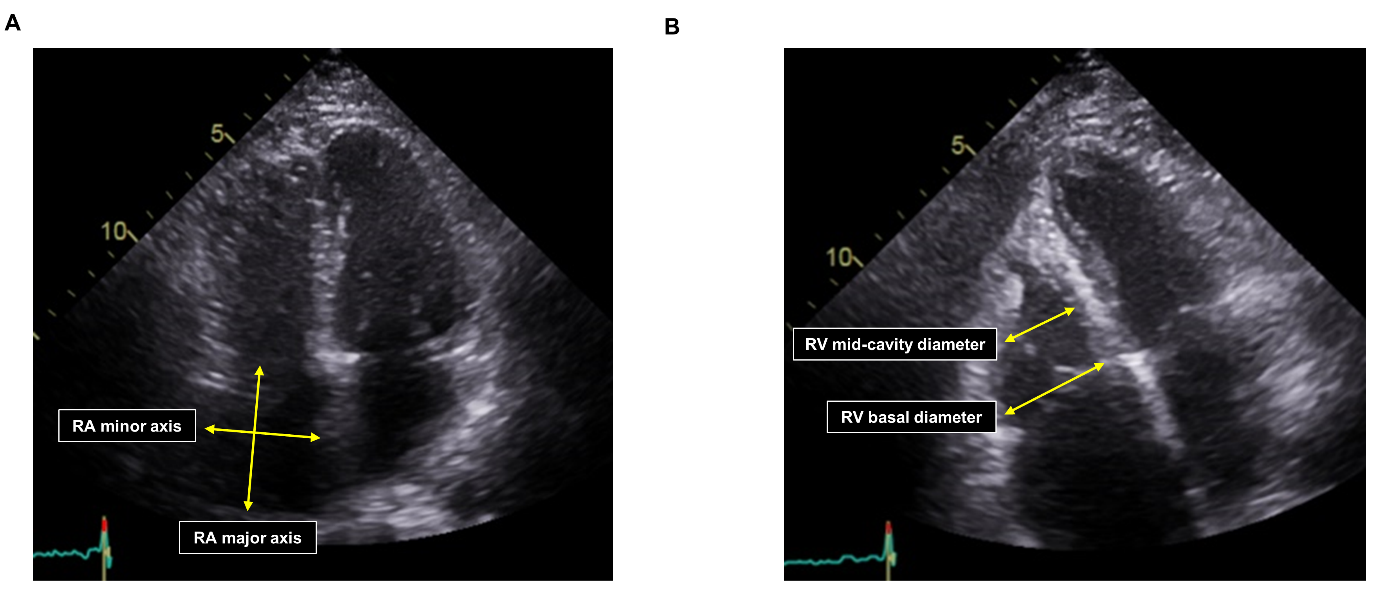


(A) RA size was determined by measuring RA minor axis and RA major axis. (B) RV size was determined by measuring RV mid-cavity diameter and RV basal diameter.

RA, right atrial; RV, right ventricular.

**Supplementary Figure S3.** Cut off value of number of attempts for left bundle branch area pacing.


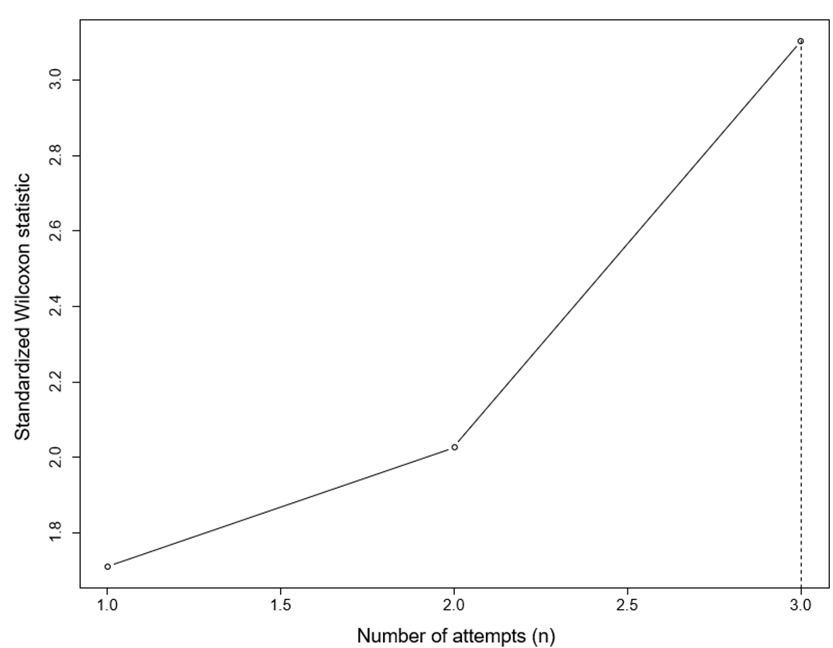


The cutoff point was calculated as 3 using the maximally selected Wilcoxon statistics.

**Supplementary Figure S4.** Right atrial minor axis dimension and final sheath size.


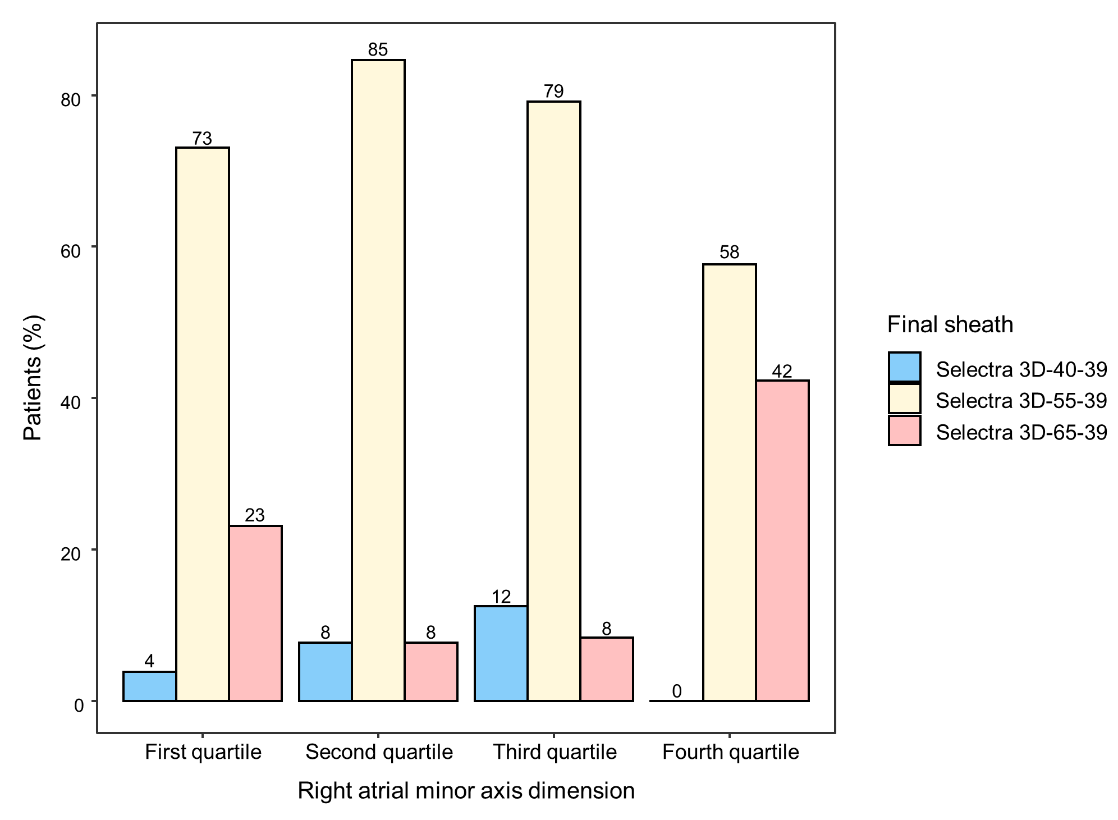


The larger right atrium needs the larger sheath for left bundle branch area pacing using stylet-driven pacing leads.

**Supplementary Table S1.** Logistic regression for predictors of success of LBBAP.

|  | Unadjusted OR  (95% CI) | P value | Adjusted OR, model1*  (95% CI) | P value | Adjusted OR, model2**^†^**  (95% CI) | P value |
| --- | --- | --- | --- | --- | --- | --- |
| **Success of LBBAP** | | | | | | |
| **Electrocardiographic characteristics** |  |  |  |  |  |  |
| Pacing indication |  |  |  |  |  |  |
| Sick sinus syndrome | 0.94 (0.86 - 1.03) | 0.20 |  |  |  |  |
| Complete AV block | 1.04 (0.97 - 1.12) | 0.26 |  |  |  |  |
| Baseline QRS morphology |  |  |  |  |  |  |
| Reference; Narrow QRS |  |  |  |  |  |  |
| RBBB | 1.02 (0.93 - 1.12) | 0.70 |  |  |  |  |
| LBBB | 1.06 (0.96 - 1.17) | 0.26 |  |  |  |  |
| Bifascicular block | 1.06 (0.92 - 1.22) | 0.41 |  |  |  |  |
| Trifascicular block | 1.06 (0.88 - 1.27) | 0.53 |  |  |  |  |
| IVCD | 0.76 (0.60 - 0.96) | 0.02 | 0.38 (0.24 - 0.61) | <0.001 |  |  |
| Pacing rhythm | 1.06 (0.90 - 1.25) | 0.49 |  |  |  |  |
| **Echocardiographic parameters** |  |  |  |  |  |  |
| LA AP diameter, mm | 0.99 (0.99 - 1.00) | 0.01 |  |  | 1.00 (0.99 - 1.00) | 0.06 |
| LVEDD, mm | 1.00 (0.99 - 1.00) | 0.33 |  |  |  |  |
| RA minor axis diameter, cm/m2 | 0.94 (0.87 - 1.02) | 0.17 |  |  |  |  |
| RA major axis diameter, cm/m2 | 0.94 (0.88 - 1.01) | 0.08 |  |  |  |  |
| RV basal diameter, cm | 0.94 (0.84 - 1.05) | 0.28 |  |  |  |  |
| RV mid-cavity diameter, cm | 0.95 (0.84 - 1.07) | 0.40 |  |  |  |  |
| **Procedural characteristics** |  |  |  |  |  |  |
| Number of screw attempts, n | 0.97 (0.95 - 1.00) | 0.04 | 0.96 (0.91 - 1.02) | 0.21 | 0.98 (0.95 - 1.02) | 0.42 |
| **Success of LBBP** | | | | | | |
| **Electrocardiographic characteristics** |  |  |  |  |  |  |
| Pacing indication |  |  |  |  |  |  |
| Sick sinus syndrome | 0.98 (0.82 - 1.18) | 0.84 |  |  |  |  |
| Complete AV block | 1.09 (0.95 - 1.25) | 0.24 |  |  |  |  |
| Baseline QRS morphology |  |  |  |  |  |  |
| Reference; Narrow QRS |  |  |  |  |  |  |
| RBBB | 0.87 (0.72 - 1.04) | 0.13 |  |  |  |  |
| LBBB | 0.90 (0.74 - 1.10) | 0.31 |  |  |  |  |
| Bifascicular block | 1.15 (0.88 - 1.50) | 0.32 |  |  |  |  |
| Trifascicular block | 1.15 (0.81 - 1.63) | 0.44 |  |  |  |  |
| IVCD | 0.82 (0.53 - 1.28) | 0.39 |  |  |  |  |
| Pacing rhythm | 0.97 (0.70 - 1.34) | 0.86 |  |  |  |  |
| **Echocardiographic parameters** |  |  |  |  |  |  |
| LA AP diameter, mm | 1.00 (0.99 - 1.00) | 0.19 |  |  |  |  |
| LVEDD, mm | 0.99 (0.98 - 1.00) | 0.07 |  |  |  |  |
| RA minor axis diameter, cm/m2 | 0.91 (0.79 - 1.04) | 0.17 |  |  |  |  |
| RA major axis diameter, cm/m2 | 0.94 (0.84 - 1.05) | 0.30 |  |  |  |  |
| RV basal diameter, cm | 0.89 (0.74 - 1.07) | 0.21 |  |  |  |  |
| RV mid-cavity diameter, cm | 0.93 (0.76 - 1.14) | 0.50 |  |  |  |  |
| **Procedural characteristics** |  |  |  |  |  |  |
| Number of attempts, n | 0.94 (0.89 - 0.99) | 0.02 | 0.89 (0.82-0.96) | 0.01 | 0.96 (0.90 - 1.02) | 0.18 |

OR, odds ratio; CI, confidence interval; RBBB, right bundle branch block; LBBB, left bundle branch block; IVCD, intraventricular conduction delay; LA, left atrium; AP, anteroposterior; LVEDD, left ventricular end-diastolic diameter; LVESD, left ventricular end-systolic diameter; RA, right atrium; RV, right ventricle; LVEF, left ventricular ejection fraction. *Model 1 was adjusted for was adjusted for age, sex, pacing indication, right subclavian venous access, simplified 9-partition method for initial lead position, echocardiographic parameters, and number of screw attempts. ^†^Model 2 was adjusted for age, sex, pacing indication, right subclavian venous access, simplified 9-partition method for initial lead position, baseline QRS morphology, and number of screw attempts.
